# Ensuring HIV care to undocumented migrants in Israel: a public-private partnership case study

**DOI:** 10.1186/s13584-019-0350-4

**Published:** 2019-11-13

**Authors:** Daniel Chemtob, Rivka Rich, Neta Harel, Nechama Averick, Eyal Schwartzberg, Israel Yust, Shlomo Maayan, Itamar Grotto, Ronni Gamzu

**Affiliations:** 1 Department of Tuberculosis and AIDS, Israeli Ministry of Health, Jerusalem, Israel; 2 Members of the ad hoc Health Committee of the Program, Jerusalem, Israel; 3 Faculty of Medicine, Braun School of Public Health & Community Medicine, Hebrew University-Hadassah Medical School, Jerusalem, Israel; 4 Division of Pharmaceutics, Jerusalem, Israel; 5 Former Head of Public Health Services, Jerusalem, Israel; 6 Former Director General, Ministry of Health, Jerusalem, Israel

**Keywords:** Migrants, HIV, AIDS, Endemic countries, Undocumented migrants, Israel, HIV care, Antiretroviral treatment, Public-private partnership

## Abstract

**Background:**

Undocumented migrants in Israel, mostly originating from HIV endemic countries, are not covered by Israel’s universal healthcare coverage. We initiated a Public-Private Partnership (PPP) to handle this public health and humanitarian challenge. The PPP venture included the Ministry of Health (MoH), pharmaceutical companies, pharmacies, and specialized HIV clinics, the Israeli HIV Medical Society (from the Israel Medical Association), and non-governmental organizations. This study describes the national policy process in conceptualizing and implementing access to HIV services for undocumented migrants through a PPP, and analyzes the preliminary results.

**Methods:**

This case study describes the process of creating a temporary Public-Private Partnership to provide HIV care for undocumented migrants based on institutional records of the Department of Tuberculosis and AIDS (DTA) and memories and reflections from partners.

This case was analyzed according to the OECD-DAC criteria for development assistance (relevance, effectiveness, efficiency, sustainability and impact). Demographic and serological data of patients referred between 2014 to 2018 were collected to monitor progress. and analyze preliminary medical and biological outcomes. Ethical approval was obtained from the Ministry of Health.

**Results:**

Creating a policy to extend HIV care to undocumented migrants was a 15 year process that confronted several challenges within Israeli and international discourse, particularly concerning governmental response to the migration crisis. The use of a PPP model involving numerous stakeholders provided a solid, local feasibility demonstration that extending HIV care as a matter of policy would have positive implications for public health in Israel. During the first 2 years of the program (2014–2015), the MoH funded medical follow-up and the pharmaceutical companies provided antiretroviral treatment (ART) free of charge for only 100 patients at any given time, in addition to ART provided by the MoH for pregnant women. Since 2016, the MoH has fully covered this service and integrated it within the Israeli health system; this constitutes the major success of the PPP program. As of December 2018, the national program has monitored 350 patients and treated 316 (90.3%). The most prevalent disease present upon referral was Tuberculosis.

**Conclusions:**

To our knowledge, this study documents the first example of a successful PPP with government partnership in a high-income country to address undocumented migrants’ lack of access to health services in general and HIV care in particular. In light of the intensification of North-South migration, this Israeli case study could be useful for other countries facing similar challenges. It also has lessons within Israel, as the country grapples with other health problems among uninsured communities.

## Introduction

Globally, migration remains a controversial topic, and health issues of migrants are often used as a pretext for discrimination [1]. This is particularly true regarding HIV/AIDS, a disease which is itself highly stigmatized.

In 2000, the United Nations declared HIV/AIDS (Human immunodeficiency virus/acquired immune deficiency syndrome) ‘a global threat’ [2]. The Global Fund to Fight AIDS, Tuberculosis and Malaria (TGF), financed by high income countries, was created to improve access to prevention and treatment for HIV, Tuberculosis (TB) and Malaria in high-endemic, low-income countries [3]. However, TGF and other international organizations do not address people living with HIV (PLWHIV) who migrate to high income countries.

Recent decades have witnessed vast waves of migration from high HIV endemic/low income countries to low HIV endemic /high income countries, primarily in Europe [4]. In 2015, 37% of all newly diagnosed HIV cases in European Union or European Economic Area (EU/EEA) countries were individuals born outside the notifying country [5].

The international response to providing healthcare to non-citizens, particularly the undocumented, varies greatly from country to country. While countries such as Belgium, France, and the United Kingdom extend HIV care to undocumented migrants, many more do not provide any services and even restrict healthcare access [6]. Of 48 responding countries in the WHO European Region, only 21 provide gratuitous ART to undocumented migrants [4]. Inability, or unwillingness, to provide care to migrant populations is often linked to national discourse and economic priorities [7]. Even in countries that offer free treatment to undocumented migrants, access to services remains problematic. Possible contributing factors include the complexities of undocumented migrants’ lives, and/or the actions of the government. For example, Grit and Spreij report that the UK purposely makes accessing health services difficult for the undocumented community as a means of discouraging permanent stay. These bureaucratic barriers are enacted despite evidence that providing ART to migrants is more cost-effective than providing treatment later [8]. Creative solutions are needed to engage stakeholders, including the government, in countries where access to care is limited for non-citizen populations.

Israel’s relationship with immigration is complex; since the establishment of the State of Israel, the government has encouraged Jewish immigration, especially in light of anti-Semitism elsewhere [9]. When non-Jewish migrants began to seek asylum in Israel, the country lacked precedent for accepting them into Israeli society including providing medical care. The lack of permanent residency prevents undocumented migrants from becoming eligible for national health insurance provided to citizens and permanent residents. In addition, the overall policy concerning undocumented migrants in Israel have been influenced by preventing permanent settlement in the past decade, which prevents extending social services to this vulnerable population, and the adoption of most inclusion solutions proposed by the MoH.

Israel is a country of low HIV/AIDS endemicity, as categorized by the WHO, with a HIV incidence remarkably lower than in most Western European countries [10, 11]. Yet, differences exist between sub-populations in Israel, and rates are sensitive to migration from countries with high HIV endemicity [12, 13].

At the end of 2013, there were an estimated 160,000 non-Israeli residents living in Israel without health insurance, equivalent to 2% of the population [14, 15]. Among them, 61,641 were people who entered Israel from Sub-Saharan Africa (SSA) between 2007 to 2012. A majority of these migrants were asylum-seekers from Eritrea and Sudan. These migrants formed 24.6% of all new HIV diagnoses in Israel in 2013. In addition, approximately 54,000 labor migrants from the former Soviet Union were reported in Israel [14]. As “undocumented” and in many cases asylum-seeking, most of these migrants do not have work permits or access to affordable healthcare [16]. In Israel, National Health Insurance cover all Israeli citizens in a way that enable access to a large basket of care. Employers of documented migrant workers need to contract an insurance that also gives access to most of these services. More generally, undocumented migrants benefit from some health services, all covered by the Ministry of Health - emergencies, pre and post maternal delivery care, and treatment of some life-threatening and infectious diseases, including TB [17].

Several international organizations have emphasized the importance of Public-Private Partnership (PPP) in low-income countries in overcoming challenges to provide affordable healthcare access, particularly in fields of HIV, TB, and Malaria [18, 19]. While governments often partner with other agencies and private companies in low-income countries, government partnership with the private sector is rarer in high-income countries [20]. In recent years, joint-commissions involving governmental and non-governmental actors in the health sector have risen in popularity in high-income countries, which indicates the recognition of the need to work across sectors [21]. However, these joint ventures are often between government and non-governmental actors, or between non-governmental actors and the private sector, rather than private-public partnerships [18].

In 1997, Israel became one of the first countries to extend Tuberculosis (TB) care to all persons, regardless of citizenship status. This program allowed a full and free of charge diagnosis, prevention and treatment of TB, both ambulatory and hospitalized based, for non-Israeli citizens. While novel at the time, the concept of extending care to non-citizens was based on fundamental human rights and infectious disease prevention and treatment principles [22]. Using the model and rationale of the existing Tuberculosis program for non-Israeli citizens in Israel, the Department of Tuberculosis and AIDS (DTA) at the MoH set out to provide HIV testing and treatment services for the undocumented community [23–26]. This paper offers a case study of how government office initiated a partnership in a PPP in Israel which ultimately increased access to ART for HIV-positive undocumented migrants. Built on the principles of the DTA to offer efficient and non-discriminatory care, the PPP successfully provided ART to a population otherwise underserved. To our knowledge, this program is a unique case as PPP in a high-income country that subsequently evolved into a government-funded program.

## Methods

This case study describes the process of creating a temporary public-private partnership to provide HIV care for undocumented migrants based on institutional records of the DTA and the memories and reflections of partners.

In addition, demographic and serological data (i.e. CD4) of all HIV patients referred to the program were collected for monitoring. The data covered for the period of the creation of the PPP in January 2014 until 2018 - 3 years following its integration into the Israeli health system (in 2016). Continuous variables were analyzed according to the mean, median, standard deviation and range. Categorical variables were analyzed according to frequencies and percentages. Student’s t-test was used to compare continuous variables while the Chi-square test was used to compare categorical variables. A *p*-value of < 0.05 was considered statistically significant.

In order to systematically analyze the strengths and weaknesses of our program, the OECD-DAC criteria for evaluating development assistance were used [27].

Ethical approval was obtained from the Ministry of Health.

## Results

### Creation of the PPP for migrant HIV care

#### Initial attempts

During the period of 1990–2006, only 2766 undocumented migrants from SSA were reported in Israel [14]. Numbers of undocumented migrants from other regions are not reported during this time. However, undocumented migrants were not eligible for public health insurance, and a system was not available to provide them with care. Modeled on the DTA’s existing TB program, the DTA sought to obtain Israeli and international funds to provide HIV care to undocumented migrants. In 2001, the DTA and Israel AIDS Task Force (IATF), a non-governmental organization that focuses on HIV prevention and treatment, applied for funding from The Global Fund (TGF). TGF is an international financing and partnership organization dedicated to investing resources to end the HIV/AIDS, TB, and Malaria epidemics globally, as called for in the United Nations Sustainable Development Goals. The organization focuses its efforts in Low and Middle Income Countries (LMIC), a majority of which are also high-endemic countries. The DTA and IATF’s request to TGF was that ART drugs for undocumented migrants from high-endemic countries residing in Israel could be purchased at the same price given to those countries. This proposal was rejected by TGF, who argued that Israel wasn’t financially eligible for subsidies [28].

The DTA next tried to collaborate with pharmaceutical companies, requesting that they provide ART free of charge, while the MoH would fund diagnosis and medical surveillance. Despite months of discussions, this endeavor was unsuccessful at that time.

In parallel, the DTA attempted to acquire ART drugs from international suppliers at the cost quoted to low-income countries, but this was rejected due to legal concerns: namely, that pharmaceutical companies that hold the drug patents would file a lawsuit.

As a result of these failed attempts, the majority of the undocumented PLWHIV were left untreated, with the exception of urgent care provided upon hospitalization, pregnant women [ 23], and a small fraction of HIV-positive migrants who received ART through the IATF, reliant on intermittent donations from pharmaceutical companies.

#### Preparatory phase, 2012–2013

By 2010, 36,616 undocumented migrants from SSA entered Israel [14]. The arrival of more than 17,000 undocumented migrants from the Horn of Africa in 2011 demanded the attention of the MoH in unprecedented terms. The increased pressure of a growing risk group and the appointment of a new Director General became crucial elements in convincing the pharmaceutical companies to partake in the joint venture. Unlike the situation in previous attempts to create a pathway to HIV care, the growing number of undocumented migrants from endemic countries contributed to a sense of urgency. The new Director General agreed that the lack of HIV care for undocumented migrants was both a public health issue crucial for Israeli health and a human rights issue. The resultant temporary partnership stipulated that the MoH would fund HIV diagnosis and medical surveillance, and pharmaceutical companies would donate ART drugs for a two-year period. In order to recruit pharmaceutical companies, the MoH committed to funding ART for at least the same number of patients following the initial 2-year period (2014–2015).

The DTA mapped the community actors who could potentially serve as partners in this venture, among both migrants and non-government organizations (NGOs). The IATF was commissioned by the DTA to perform an anthropological assessment, including mapping all formal/informal groups within the undocumented migrant population, including individuals with unofficial leadership roles and influence. Some of the HIV positive informants in the anthropological assessment subsequently became community promoters for the program. The DTA also recruited stakeholders from Israeli NGOs, which lead to partnerships with IATF and Physicians for Human Rights Israel.

The DTA asked the United Nations High Commissioner for Refugees (UNHCR) to assist in brokering an agreement to allow Israel to purchase ART at a price similar to that in the migrants’ countries of origin. The UNHCR responded that it wasn’t possible, as Israel isn’t financially eligible for subsidized prices. Concurrently, the DTA initiated discussions with senior representatives from the four Israeli Health Maintenance Organizations, and with the managerial staff of each of the 8 pharmaceutical companies which produce/import ART in Israel. Convincing the pharmaceutical company representatives was ultimately successful by holding separate meetings with each company’s representative to address individual company concerns. After the individual meetings, the MoH Director General hosted a meeting with all eight representatives, who are generally in competitive roles, to reach a final agreement. The DTA also organized two national conferences to present the program to relevant professionals and simultaneously mitigate sociopolitical concerns.

#### Implementation phase 2014–2015

The PPP partners included the DTA, who led and coordinated the program, the MoH Division of Pharmaceuticals, all regional HIV clinics, a majority of the Tuberculosis clinics, all pharmaceutical companies marketing ART drugs in Israel, one pharmaceutical chain supplier, the Israeli HIV Medical Society, and two NGOs (the IATF and Physicians for Human Rights Israel). Stakeholders met biannually to discuss the progress and obstacles of the program.

The first 40 patients accepted by the new program transferred from the IATF program, a majority of who were from SSA and arrived in Israel during the 1990s and early 2000s.

New patients were referred to the DTA by HIV clinics, hospitals, and sexually transmitted infections clinics. To be eligible for the program, patients had to have arrived in Israel at least 6 months before referral (to prevent medical tourism), have an official form of identification (asylum application, expired visa, or passport), be ineligible for national insurance, and lack private health insurance.

A medical committee composed of HIV/AIDS experts was formed to determine candidates’ inclusion in the program. Patients fell under one of four categories: 1) Approved for medical follow-up and ART; 2) Approved for medical follow-up and put on a waiting list to receive ART; 3) Approved for medical follow-up only and 4) Not accepted into the program. The committee prioritized the patient’s treatment according to ART availability, and determined the course of treatment according to clinical compatibility and medication availability.^1^

Upon acceptance into the program, participants were assigned to one of Israel’s eight HIV clinics where he/she was provided care.

ART prescriptions were filled at one pharmacy at the Tel Aviv Central Bus Station. The pharmacy was chosen due to its location at a national transportation hub and proximity to a neighborhood densely-populated by migrants. Patients were only allowed to receive medication in person with proper identifying documents. ART was provided monthly in order to monitor adherence.

All HIV patients were tested for Latent TB Infection, received a prophylactic treatment for *Pneumocystis carinii* and *Toxoplasmosis*, and all active TB cases were treated [24, 25].

Following a DTA-funded anthropological assessment of key informants in the undocumented community, a health education program was jointly created and implemented by IATF. This project was implemented by community health workers, and included health education gatherings and distribution of condoms and pamphlets in areas where undocumented migrants live. This was a means both to inform undocumented migrants about the new program and engage the population.

### Transition to national governmental program 2016-present

The major success of this PPP was its full adoption by the MoH and integration into the Israeli health system at its conclusion in 2016.

The national program was largely based on the original joint venture, with three major improvements: 1) the MoH assumed all financial costs of the program including purchase of the ART medications; 2) the budget was significantly increased which allowed more patients into the program and obliterated waiting lists; 3) Patients in the program received the ART in pharmacies operated by the hospitals in which the HIV clinics were located [26].

Table 1 displays the partners in the program of HIV care for undocumented migrants across all stages of policy development.

**Table 1:** Development of a Program to Provide HIV Care to Undocumented Migrants in Israel, 2001-present

	Initial Attempts (2001–2011)	Preparation (2012–2013)	Implementation (2014–2015)	Government Program (2016-Present)
Government Offices	DTA	DTA MoH General Director	DTA MoH General Director Pharmaceutical Division	DTA
Non-Governmental Organizations	IATF	IATF; PHRI; Israeli HIV Medical Society	IATF; PHRI; Israeli HIV Medical Society	IATF
International Organizations	TGF (refused)	UNHCR (refused)		
Health Services			TB, STI, and HIV Clinics Clalit Medical Organization Hadassah Medical Center	
Private Companies			Pharmaceutical Companies/Suppliers Tel Aviv Pharmacy	
Summary	Succeeded in extending care to pregnant women without health insurance. TGF concluded it wasn’t possible to provide ART at a discounted rate for migrants from countries that receive discounts	A change in leadership at the MoH created inter-office interests in extending HIV care. Partnerships in multiple sectors were developed. UNHCR concluded in wasn’t possible to negotiate discounted prices for ART.	Partners from multiple sectors came together to insure the feasibility of extending HIV care to undocumented migrants. Bi-annual meetings were held to insure clear communication between stakeholders	The program was fully absorbed as a government program. Services provided to undocumented migrants are now part of standard practice.

*ART* Antiretroviral treatment, *DTA* Department of Tuberculosis and AIDS, *HIV* Human immunodeficiency virus, *IATF* Israel AIDS Task Force, *MoH* Ministry of Health, *PHRI* Physicians for Human Rights Israel, *STI* Sexually Transmitted Infection, *TB* Tuberculosis, *TGF* The Global Fund, *UNHCR* United Nations High Commissioner for Refugees

### Patients’ results of program participation

Table 2 summarizes the demographic characteristics of migrants referred to the program and their inclusion status. Table 3 presents serological data for patients referred to the program. As of December 2018, 373 patients have been referred to the program, 350 (93.8%) have been monitored, and 316 (90.3%) received ART. The majority of people referred to the program originated from the Horn of Africa. Factors taken into consideration for prioritizing ART treatment were strictly medical: CD4 count and viral load, and the presence of AIDS-defining diseases and/or other opportunistic diseases. The most prevalent disease present upon referral was TB.

**Table 2:** Demographic of migrants newly referred to the program, and their inclusion status, Israel 2014–2018

Year	2014	2015	2016	2017	2018	PPP Total *2014–15*	Gov’t Program *2016–18*	Overall Total
Number referred to the program								
Female	91	18	30	22	34	109 (51.4)	86 (53.4)	195 (52.3)
Male	90	13	28	19	28	103 (48.6)	75 (46.6)	178 (47.7)
*Total*	*181*	*31*	*58*	*41*	*62*	*212*	*161*	*373*
Country of Origin								
Horn of Africa	117	22	31	19	24	139 (65.6)	74 (46.0)	213 (57.1)
Remaining SSA	41	5	17	11	17	46 (21.7)	45 (28)	91 (24.4)
Eastern Europe	9	2	10	8	19	11 (5.2)	37 (23)	48 (12.9)
Other	10	1	–	3	1	11 (5.1)	5 (3)	16 (4.3)
Unknown	4	1	–	–	–	5 (2.4)	–	5 (1.3)

*PPP* Public-Private Partnership, *SSA* Sub Saharan Africa

**Table 3:** CD4 levels of patients upon referral to program, according to year and period, Israel, 2014–2018

	2014	2015	2016	2017	2018	PPP (%)	Gov’t Program (%)
CD4 upon referral							
*0–99*	37	13	15	12	12	50 (24.5)	39 (26.2)
*100–249*	47	6	18	8	9	53 (26.0)	35 (23.5)
*250–349*	33	5	5	5	14	38 (18.6)	24 (16.1)
*350–499*	32	4	7	7	9	36 (17.6)	23 (15.4)
*> 500*	26	1	9	5	14	27 (13.3)	28 (18.8)
% CD4 < 500	85.1	96.6	83.3	87.2	75.8	86.8	81.2
CD4 - mean and SD	280 ± 192	160 ± 139	241 ± 200	260 ± 245	367 ± 292	271 ± 190	308 ± 280
Included in Program (%)	169 (93.4)	31 (100)	49 (84.5)	40 (87.2)	61 (98.4)	200 (94.3)	150 (93.2)
Received ART within year of jointing program (%)	83 (49.1)	17 (54.8)	47 (95.6)	39 (97.5)	60 (98.4)	100 (50.0)	146 (97.3)

*ART* Antiretroviral treatment, *PPP* Public-Private Partnership, *SD* Standard deviation

During the first period of implementation, the pharmaceutical companies donated ART for only 100 patients at a time. As a result, there was a waiting list, which shrank progressively as the PPP expanded into a national governmental program. Since 2017, there hasn’t been a waiting list for ART in the program.

### Program strengths and weaknesses based on OECD-DAC Critera

In order to systematically analyze the strengths and weaknesses of our program and allow for global comparison with other health development projects, the OECD-DAC criteria for evaluating development assistance were used [27]. This framework includes five criteria: relevance, effectiveness, efficiency, sustainability, and impact.

#### Relevance

The OECD-DAC defines relevance as “the extent to which the aid activity is suited to the priorities and policies of the target group, recipient, and donor.” Using this definition, the program was found relevant for both undocumented migrants and the partners of the PPP. Considering many undocumented migrants who were referred to the program had extremely low CD4 levels (Table 3), HIV care was essential to undocumented migrants in terms of improving quality of life for PLWHIV and preventing its spread in tight-knit communities. Such a situation is not unique to Israel; other researchers also described the high rate of delayed diagnosis among undocumented migrants in high income countries, with a significant percentage of AIDS-defining diseases at first presentation [29, 30]. While the program is limited to HIV care, it allows otherwise uninsured migrants some access to health services.

#### Effectiveness

Effectiveness is defined as the extent to which an aid project attains its objectives. The PPP was very effective in its objective to provide HIV care services as an intermediary step towards inclusion of undocumented migrants in the healthcare system. Before the PPP, access to ART among undocumented PLWHIV was inconsistent and very limited in scope. The PPP’s objective was to increase the number of migrants who could access ART through organized professional channels, which was achieved.

#### Efficiency

The parameters for efficiency involve three parts: 1) cost-efficiency 2) timely achievement of objectives and 3) efficiency compared to alternatives. A cost-utility analysis for the program has yet to be performed. However, the achievements of the PPP were achieved within the 2 year period agreed upon by all parties. In light of alternatives that severely limit or eliminate testing and treatment, including the prior system of only occasional provision of ART by NGOs or complete lack of treatment, the PPP and current national program are considered efficient.

#### Sustainability

Sustainability is defined by OECD-DAC as the whether the benefits of an activity are likely to continue after donor funding has been withdrawn. The PPP program was made sustainable by its absorption into the MoH as a government program, where it was not only sustained, but expanded. The PPP allowed the MoH to progressively increase the budget of government program and to ultimately sustain the entire program without the donation of ART by pharmaceutical companies.

#### Impact

Impact is defined as the intentional and unintentional changes produced by a development intervention. The PPP allowed a systematic path to HIV care for more undocumented migrants than prior to the program. However, the stakeholders were surprised by the relatively few undocumented PLWHIV who partook in the program compared to estimates of the number of undocumented migrants with HIV. While requiring further study, some of the reasons for the perceived low yield of the program may include poor access to health systems, fear from formal institutions, and continued migration beyond Israel. Considering both issues of HIV epidemiology and sociological aspects of migrant communities, the low yield did not lessen the impact of the program, which gives institutional footing to providing health services to undocumented migrants in Israel. However, this point requires consideration in building future strategies to maximize the impact in undocumented communities. In addition to more undocumented migrants accessing HIV care, the professional events surrounding the program created a framework for the importance of including undocumented migrants in care. Ultimately, the measure of impact in the HIV field is the UNAIDS 90–90-90 Care Cascade, which recommends that 90% of PLWHIV know they are HIV positive, 90% of those who know they are HIV positive are receiving ART, and 90% of those are virally-suppressed in each country [31]. In order for Israel to achieve 90–90-90 targets, they must be reached in undocumented community. Care cascade analysis will be presented elsewhere.

## Discussion

In Israel, the MoH and other stakeholders recognized the public health imperative to treat PLWHIV regardless of citizenship status. Raising the importance of the health issues in the undocumented migrant community and their socio-political ramifications was a process that took several years, numerous meetings, and multiple national conferences. The role of the government in the PPP and its transition to a government program addressed Israel’s human rights responsibilities [32].

The multi-sector partnership unified the abilities and talents of each stakeholder. The partnership with pharmaceutical companies to provide ART for a predetermined period of time without charge to the government or the recipients was a unique success of this process. In this case, IATF’s unique access and experience with this marginalized population provided the initial patient base of the PPP and an acceptable path to procuring donated medication. Additionally, the partnership with IATF contributed to trust with migrants that they would not be deported for seeking treatment.

The use of the PPP model was an essential step towards the integration of this service in the Israeli health system. While the issue of extending HIV care required a solution that addressed the financial aspects of providing care, the PPP was a successful means of garnering political support and mitigating potential concerns about the ability of the Israeli system to provide care. As a result of this process, Israel is now one of few high-income countries which provides HIV care, including ART, to undocumented PLWHIV without charge.

### Limitations

While the PPP reached a successful conclusion by its transfer to a government program, the capped number of drugs the pharmaceutical companies were willing to contribute to the program limited the amount of people who were immediately accepted for ART. While this limitation was removed after transfer to a government program, limiting patients receiving ART meant composing strict inclusion criteria for the first 2 years.

The OECD-DAC criteria were an important tool for analyzing the program. However, further study is needed to quantify several aspects of the program, both in its PPP and government program iterations. Specifically, real-life service accessibility among a politically and economically vulnerable population needs to be assessed, in addition to analysis of serological data to determine changes in transmission and adherence to treatment.

Moreover, the DTA estimates a higher number of undocumented migrants living with HIV in Israel than who were referred to the program. Analyzing potential barriers is an important next step. Researchers elsewhere have noted a lack of proof of the effectiveness of government-funded initiatives, mostly due to legal, social, administrative and economic barriers [6–8, 29, 30]. Even if migrants technically have easy access to care, many may not seek it, due to stigma, fear, and other socioeconomic disadvantages [8]. Further study is needed to know if the program in Israel enables undocumented migrants to comfortably access care, and if not, how it can be improved.

## Conclusion

This article details the process of creating a PPP to manage care for undocumented PLWHIV. To our knowledge, this is the first example of a PPP with state partnership in a high-income country to address an extreme need among the undocumented community. The PPP’s success demonstrated the feasibility of the program, and subsequently resulted in the galvanization of the program into the Israeli health system. While a PPP may not have achieved the same sustainability long-term, its use was an essential interim tool to address ideological and financial hurdles on a practical basis.

## Data Availability

The data sets used and/or analyzed during the current study are available from the corresponding author on reasonable request. The corresponding author had full access to all data in the study and had final responsibility for the decision to submit for publication.

## Footnotes

1 ART Drugs included: Atazanavir 300 mg, Darunavir 600 mg, Efavirenz 600 mg, Emtricitabine 200 mg, Lamivudine 150/300 mg, Lopinavir 200 mg, Nevirapine 200 mg, Ritonavir 50/100 mg, Tenofovir 300 mg, and Zidovudine 150/300 mg.
